# Markers and Biomarkers of Endothelium: When Something Is Rotten in the State

**DOI:** 10.1155/2017/9759735

**Published:** 2017-11-23

**Authors:** Nikolay V. Goncharov, Alexander D. Nadeev, Richard O. Jenkins, Pavel V. Avdonin

**Affiliations:** ^1^Research Institute of Hygiene, Occupational Pathology and Human Ecology, Saint Petersburg, Russia; ^2^Sechenov Institute of Evolutionary Physiology and Biochemistry RAS, Saint Petersburg, Russia; ^3^Institute of Cell Biophysics RAS, Pushchino, Russia; ^4^School of Allied Health Sciences, De Montfort University, The Gateway, Leicester LE1 9BH, UK; ^5^Koltsov Institute of Developmental Biology RAS, Moscow, Russia

## Abstract

Endothelium is a community of endothelial cells (ECs), which line the blood and lymphatic vessels, thus forming an interface between the tissues and the blood or lympha. This strategic position of endothelium infers its indispensable functional role in controlling vasoregulation, haemostasis, and inflammation. The state of endothelium is simultaneously the cause and effect of many diseases, and this is coupled with modifications of endothelial phenotype represented by markers and with biochemical profile of blood represented by biomarkers. In this paper, we briefly review data on the functional role of endothelium, give definitions of endothelial markers and biomarkers, touch on the methodological approaches for revealing biomarkers, present an implicit role of endothelium in some toxicological mechanistic studies, and survey the role of reactive oxygen species (ROS) in modulation of endothelial status.

## 1. Introduction: General Concepts

The volume of publications on the role of endothelium in health and disease has increased exponentially over the last decades, and it may seem that all problems have been thoroughly considered. There is a plethora of excellent reviews on the subject; newcomers are referred to a few of them as reliable sources of more detailed information than presented in this paper [1–4].

Endothelium is the monolayer of endothelial cells (ECs) that lines the interior surface of blood vessels, lymphatic vessels, and heart chambers. It is a natural blood container with a large surface of up to 6000 m^2^ in humans [5]. ECs form a diffuse tissue weighing around 720 g in adults, with a large proportion (over 600 g) covering the surface of capillaries [6]. The microvessels of the brain represent 3-4% of the brain compartment and constitute a significant length (ca. 400 miles) and surface of exchange (ca. 20 m^2^) between the blood and parenchyma of the brain [7]. At early stages of gastrulation, vertebrate embryos produce ECs from the mesoderm. First of all, these juvenile ECs form primitive vascular plexus de novo and later differentiate into arterial, venous, lymphatic, and capillary ECs (vasculogenesis). In the heart, the five distinct EC types (endocardial, coronary arterial, venous, capillary, and lymphatic) with corresponding phenotypes can be found [8]. The passage of plasma, molecules, and cells between the blood and surrounding tissues is normally regulated by a semipermeable barrier formed by ECs. Exchange of nutrients, respiratory, and regulatory molecules occurs in the capillaries, where the ratio between endothelial surface and blood volume is 100- to 500-fold more than in arteries and veins [1, 9]. There are vessel- and tissue-specific functions of ECs, and interacting with them is blood cells and molecules, related to differences in parenchymal and smooth muscle cells, blood oxygenation, and shear forces of the blood flow. For example, in the arteries, white (platelet-rich) thrombi are formed, while in veins, they are more fibrinous and red; in addition, veins contain valves, which often become initiation sites for a venous thrombosis. Microvascular ECs have specific phenotype in blood vessels of different organs and surrounding tissues. The well-studied endothelial morphological phenotypes are continuous (blood-brain barrier), fenestrated (exocrine and endocrine glands, gastric and intestinal mucosa, choroid plexus, glomeruli, and a subpopulation of renal tubules), and sinusoidal or discontinuous (liver, spleen, and bone marrow) [10]. ECs of the blood-brain barrier (BBB) form a continuous layer with tight junctions between the cells, lack fenestrae, and sustain very low rates of transcytosis, which significantly limits both the paracellular and transcellular exchanges of molecules [11]. Also, ECs of the BBB have low expression of leukocyte adhesion molecules, which makes it impossible for immune cells to penetrate into the healthy CNS [12]. Interestingly, alkaline phosphatase is a characteristic feature of the BBB phenotype that differentiates capillary ECs of brain from those of the periphery [7]. Moreover, the tissue-specific expression of *γ*-glutamyltranspeptidase (GGT) and monoamine oxidase (MAO) was also found in ECs of brain microvessels [13, 14]. On the contrary, thrombomodulin is absent or poorly expressed in brain microvascular and liver sinusoidal ECs, while it is abundantly present in other ECs [1]. In many tissues beyond the BBB, the transfer of macromolecules across the endothelium (transcytosis or transcellular route) is mediated by caveolae and vesiculo-vacuolar organelles (VVOs) and transendothelial channels. The density of caveolae in capillary endothelium may amount up to 10,000 per cell and greatly exceeds that in the arteries, arterioles, veins, or venules [15]. Transport of fluids and small solutes occurs in between cells (paracellular route). Clathrin-coated pits are prominent in liver sinusoidal endothelium, where they are involved in endocytosis [10]. Bone marrow ECs express E-selectin (CD62E) constitutively, whereas other types of ECs produce and express E-selectin only upon inflammatory activation. The response to inflammatory cytokines and vasoactive agents is different in various vessels, and, as a rule, the postcapillary venules' response is highly pronounced. ECs of small muscular arteries and arterioles display high angiotensin-converting enzyme (ACE) immunoreactivity in all organs studied except the kidney, while ECs of large arteries and of veins are poorly reactive or completely negative [16].

A healthy endothelium is characterized by a vasodilatory phenotype with high levels of nitric oxide (NO) and prostacyclin (PGI_2_) and low levels of uric acid and reactive oxygen species (ROS). On the other hand, ECs are involved in the aetiology of major human diseases: stroke, diabetes, insulin resistance, heart disease, peripheral vascular disease, tumor growth and metastasis, chronic kidney failure, rheumatoid arthritis, and viral infections [2]. In their quiescent state, ECs express MHC I (major histocompatibility class I) molecules and PRRs (pattern-recognition receptors) to detect PAMPs (pathogen-associated molecular patterns). Inflammatory stimuli trigger transformation of ECs to a proinflammatory and procoagulatory state. In response to inflammatory stimuli and risk factors, ECs express MHC II molecules presenting endothelial antigens to immune cells [17]. An increase in expression of other endothelial markers and cell adhesion molecules (CAM) after application of ROS or proinflammatory stimuli has also been described [18, 19]. In large amounts, ROS have a negative impact on endothelial and other cells, whereas low concentrations of ROS have signaling functions being permanently produced in cells. An imbalance in ROS production and neutralization can lead to vascular remodeling. The possible sources of ROS within endothelial and/or neighbouring cells include NADPH oxidases, mitochondria, xanthine oxidase, NO synthases, cytochrome Р450, lipoxygenases, cyclooxygenases, peroxidases, monoamine oxidases, and hemoglobin of erythrocytes [20–22].

In spite of extensive studies and impressive results, there is still a need for better understanding of dynamical changes of endothelial markers and biomarkers associated with different disorders. Also, little is known about the role of endothelium in some fields of toxicology: intoxications with organophosphates, hydrocarbons, and so forth. There is a gap between our understanding of immediate and delayed responses of endothelium and the role of ROS in these responses. It would be very interesting to reveal interrelations, feedback, and feed-forward regulatory effects of various sources of ROS in the development of vascular pathologies. Finally, great challenges exist in the methodology of biomarker discovery, which concern instrumental and computational approaches. In this review, we discuss some of these problems, which could help to re-estimate some old data as well as reveal something new in the future.

## 2. Endothelial Markers: Majors and Minors

Cell surface markers are proteins expressed on the surface of cells that often serve as markers of specific cell types. Von Willebrand factor (VWF), together with the Weibel–Palade bodies (WPB), angiotensin-converting enzyme (ACE, CD143), and the cobblestone morphology specific for monolayer cultures, was previously referred to as a few obligate criteria to confirm the authenticity and the purity of endothelial cell culture [23, 24]. A modern (though not exhaustive) list of endothelial markers with their characteristics, in short, is given in Table 1. However, the first designated endothelial markers are still under the scrupulous attention of many researchers, and great progress has been made in revealing their role, structure, and mechanistic pathways of their regulation.

### 2.1. On the Principal Markers of Endothelium

The Weibel–Palade bodies are specific endothelial organelles containing VWF, P-selectin (CD62P), and angiopoietin-2 (Angpt2), participating in platelet binding, leukocyte recruitment, and modulation of inflammation, respectively [60]. VWF is an important component of haemostasis, binding platelets at sites of endothelial damage; it is produced in megakaryocytes and ECs [61]. VWF was found in about 80% of the HUVEC cell population in culture [19]. On the surface of ECs, ultralarge VWF multimers are cut into shorter ones by the metalloproteinase ADAMTS13, binding initially to the VWF-A3 domain and then cutting individual VWF molecules at a site Tyr_1605_-Met_1606_ within the VWF-A2 domain [62]. This activity results in the generation of smaller and more globular multimers of about 20 *μ*m in length, which become detached into the circulation. The binding and cleavage sites for ADAMTS13 are buried in their round-up structure, though can be available again under high fluid shear forces [63]. Interestingly, endothelial VWF can be involved in angiogenesis. Inhibition of VWF expression by siRNA in ECs caused increased *in vitro* angiogenesis and increased VEGFR-2-dependent proliferation and migration, coupled to decreased integrin *α*v*β*3 levels and increased angiopoietin-2 release; finally, increased vascularization in VWF-deficient mice was noted [59]. In addition, a loss of VWF in ECs results in enhanced and dysfunctional angiogenesis, which is consistent with the clinical observations that in some patients with VWF disease vascular malformations can cause severe gastrointestinal bleeding [64].

Angiotensin-converting enzyme (ACE, EC 3.4.15.1), one of the principal members of renin-angiotensin system (RAS), is a СООН-terminal dipeptidyl carboxypeptidase I, converting angiotensin I to vasoconstrictor angiotensin II, degrading bradykinin [65] and amyloid beta-protein [66]. On average, only 20% of capillary ECs in each organ stains for ACE, with the exception of the lung and kidney. In the lung, all capillary ECs express ACE, whereas in the kidney, all the vasculature is devoid of ACE. In contrast to man, the rat shows homogeneous endothelial expression of ACE both in arteries and veins, but in renal vessels, ACE expression is low. The reduced ACE expression in the renal vasculature may protect the renal circulation against excess angiotensin II formation and kinin depletion, thus maintaining renal blood flow [16]. Angiotensin-converting enzyme 2 (ACE2) is a relatively new member of the RAS. It has drawn attention since 2003, when it was found that ACE2 is the receptor for SARS coronavirus and that normal levels of ACE2 in the lung are necessary for the host to combat inflammatory lung disease [67]. So the main active peptides of the RAS now include angiotensin II (Ang II), Ang III, Ang IV, and angiotensin-(1-7) (Ang-(1-7)), among which Ang II and Ang-(1-7) are the most important in health and disease [68]. Functional effects of Ang-(1-7) are different from those of AT(1) receptor stimulation and include vasodilatation, natriuresis, antiproliferation, and an increase in the bradykinin-NO (nitric oxide) system. The catalytic efficiency of ACE2 is about 400-fold higher with Ang II as a substrate than with Ang I, so this axis can be regarded as a counter-regulatory system against the ACE/Ang II/AT(1) receptor axis [69]. It has also been suggested that the Mas oncogene may function as a receptor for Ang-(1-7) [70]. The ACE2/Ang-(1-7)/Mas axis is a pathway that acts against the detrimental effects of the renin-angiotensin system. Several factors such as Akt phosphorylation, PKC activation, and MAP kinase inhibition seem to be involved in this signaling pathway. Cofilin-1, which is a widely distributed intracellular actin-modulating protein that binds and depolymerizes filamentous F-actin and inhibits the polymerization of monomeric G-actin, is involved in the translocation of actin-cofilin complex from cytoplasm to nucleus and plays a dominant role in Ang-(1-7)-induced G0/G1 arrest and autophagy in human aortic ECs [71].

VEGF receptors 1–3 contain an extracellular segment with seven immunoglobulin-like domains, a transmembrane segment, a juxtamembrane segment, a protein kinase domain with an insert of about 70 amino acid residues, and a C-terminal tail. VEGF-A stimulates the activation of preformed VEGFR2 dimers by the autophosphorylation of activation segment tyrosines followed by the phosphorylation of additional protein-tyrosines that recruit phosphotyrosine-binding proteins thereby leading to signaling by the ERK1/2, AKT, Src, and p38 MAP kinase pathways [72]. Blood vessel formation is primarily achieved by angiogenesis—EC sprouting from pre-existing vessels. Vessel networks expand when sprouts form new connections, and vessel anastomosis is spatially regulated by VEGFR1 (Flt1), a VEGF-A receptor that acts as a decoy receptor [73]. VEGFR1 modulates the activity of VEGFR2, which is the chief pathway in vasculogenesis and angiogenesis. Oxidized low-density lipoprotein (ox-LDL) impairs angiogenesis via VEGFR2 degradation and markedly suppresses HUVEC tube formation, along with induced apoptosis [74]. VEGFR3 and its ligands (VEGF-C and VEGF-D) are involved primarily in lymphangiogenesis [72].

In addition to the VEGF receptor pathway, the angiopoietin (Angpt)-Tie is another EC-specific ligand-receptor signaling pathway necessary for embryonic cardiovascular and lymphatic development. The Angpt-Tie system also controls postnatal angiogenesis, vascular remodelling, and permeability to maintain vascular homeostasis in adults. This pathway is involved in many diseases where the vasculature plays a significant role, such as in cancer, sepsis, diabetes, atherosclerosis, and so forth. Mutations in the TIE2 signaling affect vascular morphogenesis, resulting in venous malformations and primary congenital glaucoma [75]. ECs are specifically enriched for expression of Tie-2, its paralog Tie-1, the tyrosine phosphatase VE-PTP, and its ligand Angpt-2 [57]. Angpt-1 is secreted by pericytes [76]. In the quiescent vasculature, Tie-2 is phosphorylated at tyrosine residues in its intracellular domain, thus promoting barrier function and anti-inflammation. During inflammation, both rapid release of Angpt-2 from WBP and its upregulated transcription take place simultaneously; excess Angpt-2 antagonizes Angpt-1, thus reducing the signaling pathway downstream of Tie-2 [57]. It was also shown that hypoxia enhances Angpt-1 expression due to HIF2*α*-mediated transcriptional activation in pericytes [77]. At the same time, VE-PTP is upregulated in hypoxic vascular ECs by endothelium-derived Angpt-2, with subsequent negative regulation of Tie-2 [78]. Tie-2 activated by Angpt-1 stimulates Rap1 GTPase, which reduces radial stress fibres via Rac1 and nonmuscle myosin II, independent of VE-cadherin. On the other hand, Angpt1-Tie-2 can also recruit VE-PTP into EC–EC contacts, and VE-PTP dephosphorylation of Tie-2 enhances vascular permeability [75]. Moreover, activation of a VEGFR2-dependent signaling pathway causes phosphorylation of the VE-cadherin, with subsequent beta-arrestin-dependent endocytosis of VE-cadherin and disassembly of EC–EC junctions [79]. On the other hand, at EC–EC junctions, VE-PTP indirectly dephosphorylates VEGFR2 via a Tie-2-dependent mechanism; this downregulates VE-cadherin tyrosine phosphorylation and promotes EC polarity and lumen formation [75, 80]. Thus, endothelial permeability is a result of complex interplay of Angpts, VEGF, their receptors, VE-cadherin, and VE-PTP; targeting VE-PTP can stabilize blood vessels, at least in patients with a wide variety of retinal and choroidal vascular diseases [78].

Cell adhesion molecules (CAM) make up a significant group (at least a couple of dozen) of endothelial markers, which are involved in homo- or heterophilic binding with other cells or with the extracellular matrix. All representatives of the four principal protein families (immunoglobulins, integrins, cadherins, and selectins) are expressed on the surface of ECs, including IgGs [51]. Platelet endothelial cell adhesion molecule 1 (PECAM-1, CD31) is a 130 kDa protein, which is widely distributed on endothelium and hematopoietic-derived cells. It maintains the integrity of the blood vessels and therefore is involved in leukocyte-endothelium interaction and in leukocyte-transendothelial migration during inflammation [81]. As ECs are often present at inflammation sites, the cells of the BBB are involved in development and/or manifestation of Alzheimer's disease, Parkinson's disease, multiple sclerosis, some cases of bacterial meningitis, trauma, and tumor-associated ischemia. PECAM-1 and its soluble form (sPECAM-1) are potential markers and possible targets for therapies.

Inflammation modulates gene expression through the activation of NF-*κ*B and other transcription factors. Mediators of inflammation can influence BBB permeability through RLIP76 (also known as RALBP1), an ATP-dependent transporter of electrophile-glutathione conjugates [56]. The adherens junction is a principal component of intercellular adhesion and is comprised of transmembrane cadherins forming homotypic interactions between adjacent cells and cytoplasmic catenins linking the cadherins to the cytoskeleton. Inflammation promotes disassembly of the adherens junction and a loss of intercellular adhesion, creating gaps between the ECs through which diffusion of small molecules and transmigration of leukocytes take place. Adhesion of leukocytes through multiple transmembrane proteins—such as ICAM-1 (CD54), VCAM-1 (CD106), and CD47—promotes activation of small GTPases (Rac1, RhoA, and RhoG) and PTK signaling, such as activation of Src and Pyk2 [9]. ICAM-1 is one of the principal adhesion molecules, which determines changes of endothelial permeability and transendothelial leukocyte migration. Expression of ICAM-1 is increased after activation of ECs by proinflammatory stimuli; the effect of which is mediated by signaling pathways involving Akt/PKB, NF-*κ*B, МАР-kinase p38, and ERK1/2 [82]. In brain ECs, the increase in ICAM-1 expression is observed 4 hours after a stimulus and continues up to 72 hours even after a short-term application of stimulating agents [83]. ICAM-1 protein binds with integrins CD11/CD18 and LFA-1 of leukocytes, mainly neutrophils, after that they easily penetrate into tissues [84]. It is important to note that this interaction determines intensified H_2_O_2_ generation by neutrophils, that is, appearing to be the necessary condition for the formation of positive feedback. *In vivo*, ECs lose ICAM-1 from their surface (shedding), which then acts as an independent signaling agent supporting the inflammatory process in endothelium [85]. Moreover, there is a recycling of ICAM-1 after interaction with ligands: internalization, signal transduction to lysosomes, and reintegration into plasma membrane [86]. This process is controlled by PKC and Na^+^/H^+^ exchanger, which facilitate retention and/or integration of ICAM-1 in the plasma membrane of ECs [87]. Thus, studies on the regulation of endothelial phenotype and the principal signs of the state of endothelium are very interesting and hold much promise.

### 2.2. ROS and Other Modulators of the State of Endothelium

Exposure to many agents and factors determines the phenotype and life span of ECs. They respond within minutes to haemodynamic forces, vasoactive, thrombogenic, or inflammatory agents. These acute responses follow a linear receptor-mediated cell signaling pathways with influx of calcium ions, activation of phosphorylations and enzymes that generate auto- or paracrine regulators (PGI_2_, PGE_2_, endothelin-1, H_2_S, NO, CO, etc.), and recruitment and transport of vesicles containing various substances (VWF, t-PA, endothelin-1, etc.) to plasma membrane followed by apical sorting (ADAMTS13) and/or excretion [1, 88–90]. At the same time, ROS are continuously generated at low concentrations in ECs due to the transitory hyper- and hypoglycemia, hypoxia, ischemia/reperfusion, and receptor signaling pathways activated by various agonists: angiotensin II [91, 92], thrombin [93], bradykinin [94], acetylcholine [95], histamine [95], PDGF [96], TGF-*β* [97], IL-1 [98], and LPS [92, 99]. Not all of them, if any, induce generation of ROS directly, and mechanistic studies in this field have been the “hot science” of recent years. For example, LPS or angiotensin II can launch generation of ROS via monoamine oxidases A and B [92]. Among the effectors of the primary and secondary signaling agents are those molecules which generate ROS constitutively (e.g., NOXs) or casually (e.g., xanthine oxidase and NO-synthase); they exert nonlinear pleiotropic effects and can become the key factors of blood vessel pathophysiology [22]. The role of calcium in these effects can hardly be overestimated. In many cases, calcium disbalance precedes the ROS-induced dysfunction of ECs. The principal reservoirs of calcium ions are endoplasmic reticulum (ER), lysosomes, Golgi apparatus, and mitochondria. In ECs, around 75% of Са^2+^ is kept within ER and up to 25% in mitochondria [100]. Mitochondria in ECs look like a branched network being in tight contact with calcium channels of ER and plasma membrane [101]. There are 11 potential molecular sources of mitochondrial ROS [20]. Reperfusion/reoxygenation induces calcium oscillations [102], which influence the state of mitochondria and enhance generation of ROS [103], as well as exocytosis of adhesion molecules [104]. This aggravates the state of endothelium due to infiltration of leukocytes, which generate their own ROS. On the other hand, ROS can activate various calcium channels of ER and plasma membrane, leading to calcium overload of ECs: these are IP3 and ryanodine channels and some channels of TRP superfamily [105–108]. In ECs, TRPM2 (transient receptor potential melastatin 2) is regarded as the principal channels for the store-operated entry of Са^2+^ ions [109]. TRPM2 are nonselective calcium channels; their endogenous ligands are ADP-ribose (ADPr) and nicotinic acid adenine dinucleotide phosphate (NAADP), though Ca^2+^ ions and Н_2_О_2_ can potentiate activation of TRPM2 [110–112]. TRPM2 channels are considered to be sensors of oxidative stress and redox status of cells, and their activation enhances probability of cell death [113]. It was shown that extracellular buildup of amyloid-*β* (A*β*) in Alzheimer's disease (AD) impairs endothelial structure and function through activation of the TRPM2, leading to intracellular Ca^2+^ overload and vasomotor dysfunction [114]. TRPM2 is a transducer that converts oxidative stress into calcium signaling and plays an important role in ROS-coupled diseases [115]. On the other hand, exogenous H_2_O_2_ increases [Ca^2+^]_i_ and decreases transmembrane electrical resistance in lung microvascular ECs via activation of TRPV4 through a mechanism that requires the Src kinase Fyn [116]. Nevertheless, we have recently estimated that H_2_O_2_ in low noncytotoxic concentrations causes elevation of [Ca^2+^]_cyt_ in cultured HUVECs through calcium release from the two-pore channels of endolysosomal vesicles [117]. The significance of this finding is to be estimated in the near future.

РKС occupies one of the central places in the intracellular signaling and reciprocal relations between ROS and classical second messengers. This protein (a family of proteins) has structural features that render it the most important sensor of ROS and redox state of cells [118]. Various isoforms of PKC regulate assemblage and activation of Nox1-3, the necessary condition for which is phosphorylation of p47phox [119]. This subunit can be phosphorylated by all three isoforms of РKС: conventional or classical (РKС*α*, РKС*β*), novel (РKС*δ*, РКС*ε*), and atypical (РKС*ζ*) [118]. The signaling complex РKС/Nox is activated at different pathophysiological states: neurodegenerative diseases [120], atherosclerosis [121], hypertension [122], diabetes [123], and cancer [124].

Besides PKC, p47phox can be phosphorylated by many other kinases: РKА [125], MAP kinases ERK1/2 and p38 [126], casein kinase 2 (CKII) [127], AKT [128], p21-activated kinase (РАК) [129], src-kinase [130], and kinase activated by phosphatidic acid (PA) [131]. The last seems to be the mechanistic target of rapamycin complex 1 (mTORC1), which is controlled by various signals such as growth factors (including VEGF), energy status, amino acids, and mechanical stimuli [132]. PA has been shown to modulate mTOR activity through the ERK signaling pathway [133], though direct binding to FKBP12-rapamycin-binding domain of mTOR also occurs [134]. Signaling through VEGF receptors renders activation of diacylglycerol kinase alpha (DGK*α*) through its release from nucleus and subsequent production of PA from diacylglycerol (DAG) [135]. Besides p47phox, there is another way of NOX activation with some inflammatory stimuli like TNF-*α*, in which riboflavin kinase (RFK) is engaged. Binding of TNF-*α* with TNFR1 activates not only canonical NF-*κ*B signaling, but also activates NOX through RFK, which is bound to the “death domain” of TNFR1 and to the p22(phox) subunit of NOX [136].

**Figure 1:**
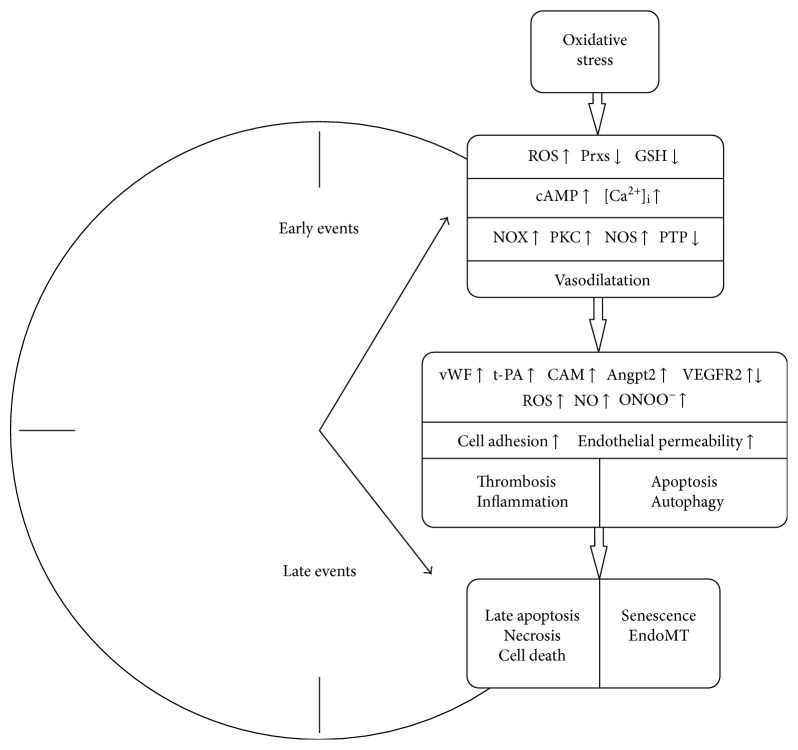
Early and late events developed in ECs upon the influence of oxidative stress. Explanations and abbreviations are given in the text.

Mechanisms of endothelium barrier dysfunction may vary depending on activating agent and prevailing or primary type of ROS. Since H_2_O_2_ is the most stable species of ROS, it is used in most experiments for modification of the redox state of the cells and studies of the signaling and toxic effects of ROS. H_2_O_2_ is produced from superoxide anion as a result of dismutation reaction, spontaneously or catalyzed by superoxide dismutases (SOD). Moreover, in endothelial and some other cells, H_2_O_2_ production is catalyzed by NADPH oxidase 4 [137]. It has been estimated that concentration of both endogenous (intracellular) and exogenous (extracellular) H_2_O_2_ can amount to as much as 500 *μ*M [138, 139]. H_2_O_2_ dose-dependently increased endothelial monolayer permeability with maximal effect at 300 *μ*M [140]. The effect of cytotoxic H_2_O_2_ on ECs relates to the depletion of the intracellular glutathione, activation of redox-sensitive kinases p38 MAP, JNK, Akt signaling involving NF-*κ*B, increased expression of aldose reductase, decreased level of sirtuin Sirt6, and enhanced expression and activity of *β*-galactosidase [141, 142]. Modified expression of eNOS (depletion) and p21 protein (increase), as well as dephosphorylation and activation of retinoblastoma proteins (Rb), have also been reported [143]. It should be noted that these studies were performed with high H_2_O_2_ doses (0.3–0.5 mM) and short periods of treatment (1–3 h). Apoptosis was shown to be a common form of the cell death [107, 142]. ECs exposed to very high H_2_O_2_ concentrations for 24 h or longer died exclusively via necrosis [144, 145]. To solve the controversy, the ratio of early apoptosis and necrosis in the HUVECs cultured close to the physiological conditions (high cell density, high serum content) was estimated after exposure to H_2_O_2_ concentrations not over 500 *μ*M. Cell viability was assessed by flow cytometry with fluorescent dyes PO-PRO-1 to detect early apoptotic cells and DRAQ7 to detect late apoptotic and necrotic cells. It was found that the primary mechanism of cytotoxic response is apoptosis. The critical concentration of H_2_O_2_, causing the cell death in a dense monolayer, is 250 *μ*M. Lower concentrations of H_2_O_2_ (up to 200 *μ*M) cause death of individual cells; however, viability of endothelial cell population is retained, and responses to calcium activating agonists do not change compared with control cells [146]. We then studied endothelial phenotype with noncytotoxic concentrations of H_2_O_2_ and found that several hours after exposure to H_2_O_2_, carbachol, or PMA, intracellular VWF level decreased, apparently due to exocytosis of WPB content [19]. This was confirmed by analysis of immunofluorescence microscopy data: H_2_O_2_ at a nontoxic concentration (100 *μ*M) increased the amount of VWF secreted by HUVECs by 43% over control and elevated total exposition of VWF on cell surface up to 94% [147]. PMA determines the progressive increase in expression of ACE (CD143), by 42 and 180% after 3 and 24 hours, respectively, whereas under the action of H_2_O_2_, ACE expression increases slowly and insignificantly (maximally by 34% after 24 hours of 200 *μ*M H_2_O_2_ application). ACE molecules are presynthesized and stored in endothelial cells, and their expression in plasma membranes depends mainly on PKC, which also controls shedding of ACE from the cells [148]. Under exposure to PMA or H_2_O_2_, the most significant change was in CD54 expression, which started to increase even at 15 min of PMA application (38%), with its expression increased more than fivefold 3 h after exposure and maintained at this level for 24 h. The effects of H_2_O_2_ were not so impressive, with expression of CD54 being the most pronounced: 1 day after exposure to 100, 200, and 300 *μ*M of H_2_O_2_, CD54 expression increased by 61, 126, and 255%, respectively [19]. The next most manifest response was the level of expression of CD309 (VEGFR-2), which is known to increase endothelial permeability in microvascular bed [45]. The level of VEGFR-2 was decreased in ox-LDL-treated HUVECs due to overproduction of ROS, and this was coupled with apoptosis by Annexin V-FITC staining and increased caspase-3 activity [74]. In our experiment, the level of expression of CD309 increased by 60–100% within 24 h after the stimulus application, and the effects of PMA and H_2_O_2_ were similar [19]. It is known that CD31 molecules (PECAM-1) mediate binding of leukocytes and help them to penetrate into tissues [149]. Importantly, all used concentrations of H_2_O_2_ caused CD31 expression to increase 2-3 times higher in 24 hours than after exposure to PMA. This may indicate that PECAM-1 expression is a result of synergistic activation by H_2_O_2_ of several signaling pathways, such as NF-*κ*B, Akt/PKB, MAP-kinases p38 and ERK1/2, and so forth. The changes in CD106 and CD29 expression were generally insignificant; at all concentrations of PMA or H_2_O_2_, an increase of 16–24% in expression of these antigens was observed after 24 h [19]. A combined (simultaneous or consequent) increase in expression of surface antigens of ECs under oxidative stress certainly determines the development of vascular dysfunction and characteristics of pathological and critical states (Figure 1).

### 2.3. Endothelial Cell Senescence and Endothelial-Mesenchymal Transition Process

Most mitotically competent mammalian cell types can react to oxidative and other stress factors by undergoing a phenotypically distinctive form of growth arrest called “cellular senescence.” In endothelial cells, these changes result in a phenotype that is proinflammatory, proatherosclerotic, and prothrombotic [150]. Two types of cell senescence have been identified: (1) replicative senescence with telomere attrition and (2) stress-induced premature senescence without telomere involvement. Both types of cell senescence lead to arrest of endothelial cell growth and loss of vascular functions, thus contributing to development of cardiovascular diseases [151]. Activation of renin-angiotensin-aldosterone and endothelin systems causes endothelial dysfunction, vascular remodeling, and endothelial senescence by inducing ROS production and promoting inflammation and cell growth [152]. In addition, there are changes in the expression and plasma levels of important endothelial components related to endothelial-mediated modulation in hemostasis. These include alterations in the metabolism of nitric oxide and prostanoides, endothelin-1, thrombomodulin, and VWF. These alterations potentiate the procoagulant status developed with aging, highlighting the endothelial role in the development of thrombosis in aging [153]. It was demonstrated that sirtuins SIRT1, SIRT3, and SIRT6 can protect ECs against vascular aging [151].

Sequentially or in parallel with senescence, and under chronic oxidative stress, inflammation, influence of insulin-like growth factor II, transforming growth factor-*β* (TGF-*β*), and so on, ECs may contribute to fibrosis through the process of endothelial-to-mesenchymal transition (EndoMT) [154–156] (Figure 1). It is a process through which certain subsets of ECs lose endothelial characteristics and transform into mesenchymal-like or smooth muscle-like cells, susceptible to being redifferentiated into mesodermal cell types, including osteoblasts, chondrocytes, and adipocytes [157]. Initiation of transcriptional reprogramming is led by the EndoMT-transcription factors Slug, Snail, Twist, and Zeb1/2; this event is coupled with loss of apical-basal by polarity and severance of intercellular junctions. Importantly, these changes persisted after removal of the inducing agents and were accompanied by functional loss of acetylated LDL uptake and migratory capacity and acquisition of de novo collagen synthesis capacity [158]. It is still unclear what regulatory signals determine these cells to undergo complete or partial EndoMT. In the case of sprouting angiogenesis, the contact-dependent Notch signaling pathway plays a role in this process [159]. EndoMT also exists and contributes to the development and progression of cardiac fibrosis, lung fibrosis, liver fibrosis, corneal fibrosis, and so forth.

TGF-*β* binding with subsequent activation of Smad-dependent and Smad-independent TGF-*β* intracellular signaling is considered to be the most important pathway for initiation of EndoMT [160]. Enhanced expression of NOX4 caused by TGF-*β* results in Snail1-mediated EndoMT. In addition, ET-1 synergistically stimulates EndoMT induced by TGF-*β*, with the canonical Smad pathways involved. Hypoxia is another factor inducing EndoMT via HIF-1*α* activation of Snail1. A plethora of data suggest that Snail1 is a crucial regulatory molecule in EndoMT. The level of Snail1 is regulated by GSK3-mediated phosphorylation, so that phosphorylated Snail1 undergoes proteasomal degradation. Cav1 plays an inhibitory role through the internalization of TGF-*β* receptors with their subsequent degradation. EndoMT can be also modulated by morphogen pathways including Wnt, Sonic Hh, and Notch. The final result of these intracellular signaling events is the activation of a mesenchymal cell-specific transcriptional gene regulation program leading to the enhanced synthesis of various myofibroblast-specific and profibrotic macromolecules including *α*-SMA, COL1, COL3, FN, COMP, and the MMP-inhibitor TIMP. Simultaneously, the repression of EC-specific gene products such as CD31/PECAM-1, VE-cadherin, and VWF occurs, leading to the phenotypic conversion of EC into myofibroblasts, which are mainly responsible for the fibrotic process [160].

miR-20a has been shown to be reduced during EndoMT, and restoration of its expression by application of FGF-2 correlated with EndoMT regression [161]. In contrast, miR-21 is expressed downstream of TGF-*β* and activation of TGF-*β* signaling leads to its overexpression and the appearance of EndoMT. Importantly, blockade of endothelial miR-21 expression reduced EndoMT [162]. These two miRNAs, thus, act, respectively, upstream (miR-20a) and downstream (miR-21) of activated TGF-*β* signaling [163].

TGF-*β* is considered to be a major mediator of EndoMT, whereas bone morphogenetic protein 7 (BMP-7, also known as osteogenic protein-1 or OP-1) and fibroblast growth factors (FGFs) are reported to augment or oppose TGF-*β*-driven EndoMT in specific contexts [164]. Also, stimulation of endothelial autophagy seems to be promising in reduction of EndoMT [165]. Autophagy inducers, rapamycin and trehalose, counteracted the EndoMT process triggered by TGF-*β*2 by decreasing the phosphorylation level of Smad3 and reduced the expression of Snail [166]. A breakthrough discovery was published in 2014 demonstrating the ability of cardiac fibroblasts adopt an endothelial-cell-like phenotype after acute ischemic cardiac injury [167]. Fibroblast-derived ECs exhibited anatomical and functional characteristics of native ECs, and induction of the p53 pathway in cardiac fibroblasts augmented mesenchymal-to-endothelial transition and improved circulation and cardiac function.

To reduce the EndoMT in systemic sclerosis-associated interstitial lung disease, cyclophosphamide is administered by clinicians, whereas mycophenolate or methotrexate has been used for less severe skin progression. Novel agents capable of modulating fibrotic and inflammatory pathways involved in systemic sclerosis pathogenesis include tocilizumab, pirfenidone, tyrosine kinase inhibitors, lipid lysophosphatidic acid 1, and NOX4 inhibitors [168]. As for natural compounds, geniposide (an iridoid glycoside isolated from the gardenia plant) and glycyrrhizin (a saponin of *Glycyrrhiza glabra* or liquorice root) seem to be promising. Geniposide remedied bleomycin-induced dermal capillary loss and fibrosis in mice; the expression of key EndoMT factors (Slug, Snail, and Twist) and the mTOR signaling pathway (mTOR and S6) were also attenuated by geniposide treatment [169]. Glycyrrhizin significantly ameliorated dermal fibrosis in bleomycin-treated mice, which was partly attributable to blockade of TGF-*β* signaling in dermal fibroblasts through the downregulation of thrombospondin 1, a latent TGF-*β* receptor, and transcription factors Smad3 and Ets1. Furthermore, bleomycin-dependent induction of T helper type 2-skewed immune polarization, M2 macrophage infiltration, and EndoMT was greatly suppressed in mice administered glycyrrhizin [170].

## 3. Seeking Biomarkers, Endothelial Wanted

The purpose of diagnostics is to determine the state of a patient, which is characterized by a change in the complex of biochemical, immunological, and other indicators (biomarkers). The term “biomarker” is generally used to define any indicator reflecting the state of an organism as a result of its development and functioning, as well as interaction of the body with an external factor of a chemical, physical, or biological nature [171]. Biomarkers are defined as the measurable characteristics (biochemical, physiological, and immunological) of an individual that may be considered as risk factors for a disease or outcome, or as indicators of disease progression or of treatment-associated changes [172]. There is no sharp boundary between the markers and biomarkers of endothelial cells, since some of them are not only expressed at the surface, but cut off the cells (shedding) to the circulation (ACE, VWF, ADAMTS-13, soluble adhesion molecules, etc.). Inflammation is indicated by elevated levels of sVCAM, sICAM, E-selectin, along with C-reactive protein (CRP), TNF-*α*, and other inflammatory cytokines. ECs, their progenors and derivatives, can be biomarkers per se. Normally, the population of endothelial progenitor cells (EPCs) is high, whereas levels of endothelial microparticles (EMPs) and circulating endothelial cells (CECs)—indicative of endothelial damage—are low [2, 4]. EMPs are circulating submicron-sized vesicles originated from damaged ECs that have various biological functions. They have been shown to act as primary and secondary messengers of vascular inflammation, thrombosis, vasomotor response, angiogenesis, and endothelial survival. EMPs are emerging as biomarkers of dysfunctioning endothelium, whereby differential presence of EMPs is linked to disease manifestation [173]. Other phenotypic characteristics of a dysfunctional endothelium include impaired vasodilation, increased lipid peroxides, uric acid, nitrotyrosine and NO, a procoagulant and proinflammatory phenotypes with decreased vascular repair capacity, and increased numbers of EMPs and CECs [2, 4]. However, there are examples of endothelial biomarkers that deserve special consideration.

### 3.1. Transaminases

Transaminases are traditionally considered to be markers of the liver and heart, while the contribution of the endothelium to their blood level is rather obscure. In the ischemia-reperfusion injury, endothelial permeability is increased, together with neutrophil sequestration and a 4-fold rise in blood transaminases [174]. Activated macrophages can generate a significant amount of IL-1*β*, which causes damage to the lung tissue and is also coupled with elevated level of transaminases in blood [175]. A significant clarification of the impact of endothelium on the dynamics of blood transaminases has been made through studies of Mediterranean spotted fever (also known as Marseilles fever and Boutonneuse fever). In infected patients, an elevated level of transaminases (up to 3-fold above the norm) is observed in the blood, which is usually associated with damage to the liver or heart [176, 177]. In some patients, increased levels of transaminases in the liver and spleen were also registered, despite the fact that there was no chronic liver or heart disease which could be a source for transaminases. An experiment with infection of ECs with *Rickettsia conorii*, the bacterium of Marseilles fever, showed a significant increase of transaminases in the supernatant, along with specific endothelial markers [178]. It is interesting to note here that elevated levels of triglycerides, apoB, *γ*-glutamyl transferase, and alkaline phosphatase and low levels of cholesterol, apoA, and blood platelets were also observed in some patients, whereas bilirubin, albumin, and *γ*-globulin were at normal levels, indicating that the state of the liver was normal, though the state of endothelium was compromised. Endothelium is the site of action of three principal lipases (lipoprotein lipase, hepatic lipase, and endothelial cell lipase), which play a central role in triglyceride and phospholipid hydrolysis [179]. It should be noted that *γ*-glutamyl transferase and alkaline phosphatase are also biomarkers of endothelial damage [7, 180]. In heart stroke, an elevation of transaminases, together with “classical” endothelial markers ICAM-1, endothelin, and VWF, and decreased level of ACE were observed [181, 182]. Systemic inflammatory diseases are usually coupled with elevated levels of biomarkers of endothelial injury, together with biomarkers of dysfunction of various organs. However, due to the lack of investigations covering all these and other biochemical parameters of blood, one cannot conclude on the specificity and importance of an indicator [183].

### 3.2. Endothelium and Toxicology of Organophosphates

**Figure 2:**
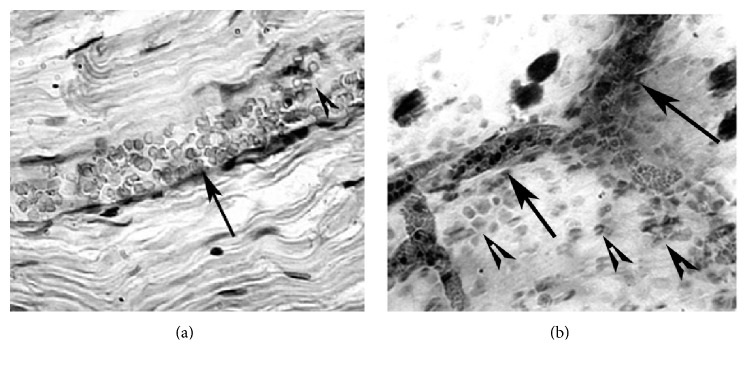
(a) Dilated venule (arrow) within the endoneurium of sciatic nerve with aggregated blood cells and their penetration (diapedesis) through the vessel wall (arrowhead). Three weeks after acute exposure to soman (GD). H&E, ×350. (b) Distonic venules (arrows) and congestion of blood cells within (mostly erythrocytes) and around (mostly granulocytes and macrophages, arrowheads) in the mesenterium of small intestine. Two weeks after subacute intoxication with VR (Russian Vx). H&E with additional benzidine staining, ×150.

**Figure 3:**
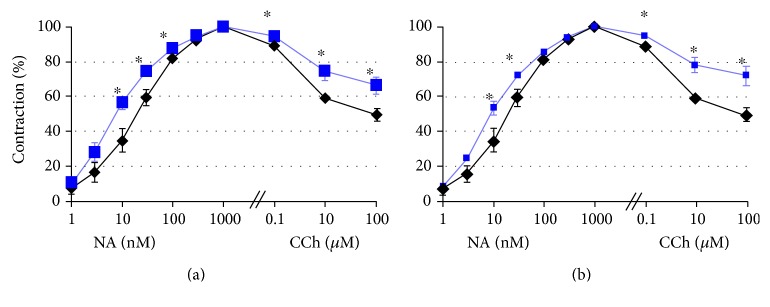
Norepinephrine- (NA-) induced contraction and carbachol- (CCh-) induced relaxation of rat aorta after subchronic (3 months) exposure to paraoxon (a) and DFP (b) in doses 1/100LD50 (black diamonds) and 2 months of “rehabilitation” period (blue squares). ^∗^*p* < 0.05.

Much more complex and obscure is a role of endothelium in the development of effects after chronic or acute exposure to some toxic agents, most notably organophosphates (OPs) or nerve agents. Not only neuronal and neuromuscular synapses but also ECs have many signs of autonomic cholinergic regulation. Due to muscarinic receptors, ECs became famous in 1980 as the site for generation of endothelium-dependent relaxing factor (EDRF, later revealed to be nitric oxide) [184]. However, N-cholinoceptors have also been found [185], as well as activities of acetylcholinesterase (AChE) [186, 187], cholinacetyl transferase providing synthesis of acetylcholine, and the vesicular system of acetylcholine transport out of the cells [188, 189]. In spite of recent advances in the therapeutic treatment of OP poisonings, they usually proceed with predominance of a heavy cholinergic crisis, and even in the case of survival of a victim, delayed pathologies are often observed: the so-called “intermediate syndrome”; OP-induced distal sensorimotor axonophaty; symptoms of vegetative changes of the circulatory system; the CNS “microorganic disorders” with unknown ethiology, and so forth [190–192]. Because AChE is the main target of OPs, the principles of existing therapy of acute poisoning are converged to elimination of effects of AChE inhibition by OPs, that is, administration of cholinolytics (e.g., atropin), reactivators of AChE (oximes), and anticonvulsant drugs (agonists of GABA- and antagonists of glutamate receptors); as a preventive means before the alleged poisoning, reversible AChE inhibitors can be used (e.g., pyridostigmine) [193, 194]. Mechanistic studies are reduced to revealing interactions of OPs with molecular targets in nerve cells. Thus, it was estimated that OPs can interact with covalent binding to tyrosine residues of tubulin, and the OP-binding sites are dislocated near the GTP-binding sites or within loops interacting with protofilaments [195], and this could partly explain a decay of fast axonal transport and neurophysiological effects of chronic exposure to OPs [196, 197]. Just a few papers report an influence of OPs on endothelium. In an experimental model in mice that examined the pulmonary effects of O,O,S-trimethyl phosphorothioate (OOS-TMP), injury of ECs was accompanied by significant enhancement of wet lung weight and percentage of lung water content [198]. In another study, chlorpyrifos over a wide concentration range showed no effect on isolated thoracic aorta, though plasma AChE activity was decreased, while LDH, ALT, GGT, and AST activities, together with plasma NO level, were increased in the chlorpyrifos group [199]. In our studies, we also paid attention to damage of the vascular endothelium in development of OP-induced delayed pathology [192, 200, 201]. We were the first to suggest that thrombotic microangiopathic disorders, resulting from endothelial damage, could be the primary reason for development of delayed effects of OP intoxication; the significance of this kind of disorders has been re-estimated over recent years [3]. An interrelation of neural blood flow and neuromuscular conduction is of great interest taking into consideration the low percentage of endoneural capillaries. A clue for dependence of the trophism of nerve fibres upon the vasculature comes from the following data: the volume of capillaries does not exceed 4% of the total volume of nerve fibre [202]. In addition, there are no lymphatic vessels within the peripheral nerves, which could provide an effective outflow of the capillary infiltrate and prevent development of edema [203, 204]. Earlier, we suggested a role of nonsynaptic and blood vessel-dependent mechanisms of delayed effects under acute and subchronic influence of OPs. Several weeks after exposure to OPs, we found angiodistonic disturbances, new or opening of pre-existed arteriovenous anastomoses, dilated and distonic venules within the endoneurium of sciatic nerve, and mesenterium of small intestine (Figure 2). The morphological signs were accompanied with functional ones, such as norepinephrine-induced contraction and carbachol-induced relaxation of rat aorta after subchronic (3 months) exposure to paraoxon and DFP in doses 1/100LD50 and 2 months of “rehabilitation” period (Figure 3). The morphological and functional changes of the microvessels can affect the functional state of platelets, creating an etiology of delayed effects of acute and subchronic toxicity. The observed changes reflect multiple microangiopathies, so that obvious clinical signs are often not seen due to various compensatory mechanisms.

MS/MS analysis of blood plasma of animals intoxicated with RVX revealed fragments of fibrinopeptide A, signifying that exposure to RVX caused inactivation or reduced expression of exopeptidases (aminopeptidases) [192]. Various aminopeptidases are known to function in blood plasma and the vascular bed, which could be responsible for these effects. Aminopeptidase A (L-alpha-aspartyl-(L-alpha-glutamyl)-peptide hydrolase, EC 3.4.11.7) hydrolyses acidic amino acids (Asp and Glu in the fragments of fibrinopeptide A) [205]. Aminopeptidase N (CD13/APN, EC 3.4.11.2) is a multifunctional enzyme and the principal aminopeptidase in the circulation; it is expressed on cells of myeloid origin, including monocytes and neutrophils [206]. It was also found on fibroblasts and epithelial and endothelial cells; angiogenic growth or signaling factors were shown to increase the CD13/APN expression [207–209]. On the other hand, alphastatin, a 24-amino acid peptide derived from the amino terminus of the *α*-chain of human fibrinogen, possesses an antiangiogenic activity that was previously revealed in fibrinogen degradation product E [210]. Many other proteolytic degradation products were also revealed as endogenous inhibitors of angiogenesis [211].

### 3.3. New Methodological Techniques for Identifying New Endothelial Biomarkers

Biomarkers can be used, and ideally should be used, for quantitative evaluation of the body's response to external effects [171]. Quantitative characteristics include the diagnostic sensitivity, specificity, predictive value, likelihood ratio, and so forth. An ideal biomarker is characterized by a high sensitivity, specificity, and predictive value; it is reproduced in humans of different sexes and ethnic groups, and the procedure for its determination is cost-effective. However, rarely does such a marker stand alone as a single parameter; rather, it is a derivative of several original indicators [212, 213]. At the same time, a complex of physiological and biochemical methods combined with an appropriate analytical platform should be relatively simple (low-invasive and noninvasive methods), universal (modular), and flexible (algorithmic). Ambiguous expression pattern and complexity of determining many biomarkers decrease their predictive value, leading to an overdue diagnosis and bad prognosis. Parallel measurement of multiple “early” biomarkers would certainly increase the diagnostic accuracy. Together with identification studies, validation studies of multimarker assays are urgently needed.

A biopsy is the gold standard in diagnosing many chronic diseases, though morphological methods are not a common tool in clinical diagnostics. Due to the invasiveness, these techniques are not suitable for continuous monitoring. Moreover, many diseases may not have a morphological analog of the “gold standard,” which necessitates the permanent expansion of functional and point-of-care diagnostics in clinical practice [214, 215]. The search for safe, reliable, and inexpensive methods has resulted, for example, in the development of a large number of biomarkers for noninvasive assessment of liver fibrosis representing both simple, directly measurable parameters and their derivatives (indices or ratios), which increase the sensitivity and/or specificity of assessment (diagnosis): the ratio of the level of AST to the level of ALT—the so-called De Ritis ratio; the ratio of AST to the number of platelets; PGA index—the ratio of prothrombin time and the level of GGT and apolipoprotein A1 (the PGA index was later modified due to *α*-2-macroglobulin and became known as the PGAA index); and the FibroTest, known as FibroSure in the US, including *α*-2-macroglobulin, apolipoprotein A1, GGT, haptoglobin, and total bilirubin [216].

Recently, we reported on new screening indicators (biomarkers) for health assessment of the personnel at a chemical weapon destruction facility (CWDF). Blood plasma of healthy donors and of CWDF personnel occupied in different facility workshops or zones was analysed for 27 cytokines. A significant elevation of eotaxin concentration was revealed in blood plasma of the “dirty zone” personnel. For screening assessment of health state of the personnel, a new complex biomarker was suggested: relation of eotaxin × IFN-*γ*/TNF-*α*, capable of differentiating the “dirty zone” personnel from others with a sensitivity of 67.9% and a specificity of 87.5% [213]. The differences between the control group and personnel occupied in “clear” and “conditionally dirty” zones in the CWDF are much greater, as compared with the well-known and popular index IFN-*γ*/TNF-*α* characterizing a balance of T-helpers (Th1, Th2) [217]. Importantly, for our subject, IFN-*γ* can directly influence the level of eotaxin-1 that is produced by ECs, whereas TNF-*α* can influence the level of eotaxin-1 that is produced by monocytes [218, 219]. Significant increase of the index is a sign of reciprocal and even ontological relations of IFN-*γ* and eotaxin, which provide a “synergistic effect” for diagnostics.

A combination of markers defines a combinatorial biomarker, whose identification usually involves multivariable assays [220]. A combinatorial biomarker provides a specific pattern that bears much more information than just individual markers. The detection of these patterns requires the complex bioinformatics analysis that is necessary for all multidimensional data. However, the expression of these biomarkers, as well as their prognostic and predictive value in a combination, needs to be well-defined. However, even a search process presents great difficulty when it deals with a high-dimensional dataset and a huge number of parameters [221]. An example of this approach is the research in neurology related to the biochemical diagnosis of cerebrovascular disorders. To diagnose a particular subtype of stroke, assess its severity, and predict possible development and the probability of a lethal or successful outcome by biochemical criteria, various authors have attempted to simultaneously measure up to 50 indicators and search for correlations between them. Ultimately, a set comprising four or five of the most reliable indices (markers) was found, which taken together provided a sensitivity and specificity of over 90% [222–224]. The strategy of these studies was to combine sensitive but not tissue-specific markers with at least one marker specific to the CNS. The use of nonspecific markers is required in order to increase the sensitivity of the complex test. Biochemical tests have an undeniable and fundamental advantage over other complex instrumental methods of diagnostics; they can be performed as point-of-care testing [224].

One of the fields of the search for new biomarkers is metabolomics. The metabolome is the end product of the genome; in fact, it is the totality of all the metabolites, predominantly of low molecular weight. Metabolites are the final or intermediate products of metabolism of living systems (biological matrices) characterizing their functioning mode. In an epistemological classification, the transcriptome (the totality of all mRNAs) and the proteome (the totality of all proteins) should be placed between the genome and the metabolome. The study of the metabolome, however, is sufficiently rewarding, firstly, because the major metabolic pathways are fairly well studied and the existence of the majority of metabolites is well known and, secondly, because the total number of metabolites is relatively small (3 × 10^3^) as compared to the total number of modified proteins (over 10^7^) [225]. It should be noted that metabolomics deals not only with biomarkers (e.g., metabolites of endogenous origin). In toxicological and pharmacological studies, metabolites are divided into endogenous and exogenous [226]. Metabolites of foreign substances (drugs, poisons, etc.) are called xenometabolites, or xenobiotics [227]. For this field of study, there is another term—metabonomics [228]. Alternatively, biomarkers can be divided into three categories: biomarkers of exposure (concentrations of chemicals, their adducts and derivatives in the body fluids or tissues as a result of external or internal exposure); biomarkers of effects (early and delayed biochemical and clinical effects); and biomarkers of susceptibility (individuals with increased sensitivity towards target molecules) [229, 230].

The main methods of metabolomics are nuclear magnetic resonance spectroscopy (NMR) on different nuclei (^1^H, ^13^C, ^31^P, etc.) and gas chromatography-mass spectrometry (GC-MS). NMR spectroscopy is used to detect low molecular weight metabolites in blood, urine, serum, and tissues [231]. The advantages of NMR are the simplicity of sample preparation and spectrum interpretation, as well as high performance. The main drawback of NMR is low sensitivity, which is only sufficient to determine those metabolites whose concentrations are relatively high. High-performance liquid chromatography with mass-selective detection (HPLC-MS) is much more sensitive to low molecular weight (3000 Da) metabolites and even peptides. Its precision makes it possible to accurately identify many peptides and nucleosides even in complex matrices [225]. However, HPLC-MS has many limitations in detecting some low molecular weight compounds, for example, fatty acids which are essential components of metabolism. The primary data are processed using hierarchical multivariate curve resolution (multivariate profiling), which includes principal component analysis (PCA), orthogonal projections to latent structures (OPLS), and partial least square discriminant analysis (PLS-DA) [232, 233]. These methods are a part of chemometrics, an independent scientific direction that originated in the 1970s at the intersection of mathematics (informatics) and analytical chemistry. It is important to emphasize that the methods of metabolomics are not intended to replace the methods of classical biochemistry (spectrophotometry, enzyme immunoassay, etc.). Metabolic profiling provides a unique opportunity to perform diagnosing and monitoring at a qualitatively new level only in conjunction with the methods of biochemical analysis [212].

In our recent studies, the influence of chronic exposure to low doses of C_6_-C_10_ aliphatic hydrocarbons on metabolic profiles of rats was investigated using GC-MS and high-resolution LC-MS, then a chemometrical algorithm, was applied and combinatorial biomarkers were derived. The ratio of concentrations of pyrophosphate (РР_i_) and oxalate in the blood plasma was found to be the most sensitive biomarker, called the “*pyrophosphate index*,” whose changes were statistically significant in all groups, even in that exposed to the lowest dose of 5.2 mg/m^3^ [234]. Having analysed a broad range of metabolites, we came to the conclusion that increased level of РР_i_ in blood plasma is evidently caused by a decreased level and/or activity of tissue-nonspecific alkaline phosphatase (TNAP), which is a marker of the BBB endothelial cells [7]. This indicates a risk of hypophosphatasia and neurological disorders, including epilepsy and disruption of myelin synthesis [235]. It can also be coupled with an increase of phosphorylated tau protein [236] and upregulation of FAT/CD36, a phosphorylated fatty acid translocase [237]. In addition, another important index was revealed; an elevation of hypotaurin/taurin indicates abnormality in the mechanisms of utilization of Cys and may lead to deficit of glutathione, since Cys is a source of both hypotaurine and glutathione. Moreover, having analysed a broad range of related metabolites, we suggested a “*redox hypothesis*” of pathogenesis of toxic polyneuropathy, as an alternative to the long-established “*energy hypothesis*” [238]. The redox hypothesis covers not only the energy aspects, but also implies a violated interface between energetic, signaling, and metabolic processes. In this sense, it conforms to the principles of “orthogonal regulation” of the signaling and metabolic processes in living cells, including endothelial ones [239]. It seems that the endothelium due to its difuse and strategic position provides an orthogonal control of other states, namely morphofunctional states of organs and/or other tissues (e.g., neural).

## 4. Concluding Remarks and Future Directions

Endothelium is strategically located at the interface between blood and interstitial tissues, and ECs exist in a dynamic equilibrium with their environment constituting simultaneously a source, a barrier, and a target of defensive mediators and therapeutic interventions. As a rule, vascular pathology is caused by multiple factors, involving genetic and environmental ones, whereas the current clinical treatments mostly target individual molecules involved in the pathophysiological pathways; this approach to genotype/phenotype studies is governed by the “one-gene/one-phenotype” hypothesis. At the same time, science is moving ahead and the combination of genomics, proteomics, and metabolomics has described the origin (etiology) and essence (pathophysiology) of vascular-dependent pathologies in a big-data fashion and also revealed new and unrivalled relationships between the omic variability and the restricted definitions of clinical phenotypes of vascular disease [240]. The phenomics strategy was announced about two decades ago [241, 242], which gradually changed the conventional phenotype/genotype/genome approach to a new systematic phenome/genome/proteome study. A phenome is the whole set of an organism's phenotypic traits as a result of the expression of genome and specific environmental influence. Phenomics is a novel approach to quantitatively correlate multiple phenotypic traits to variabilities in transcriptome, proteome, metabolome, interactome, and environmental factors, together with genome. The development of phenomics will not only define a set of biomarkers for diagnosis of vascular pathology, but also reveal novel targets for combination therapy, as well as novel testing systems for animal-free chemical safety assessment, thus making a revolutionary paradigm shift in the preclinical testing of drugs and clinical treatment of these devastating diseases [240, 243]. In parallel, very similar concepts of toxicometabolomics have developed, known as “toxicity pathways” and “toxicity signatures.” Toxicometabolomics is intended to integrate our understanding of the toxicokinetics of chemicals and the toxicodynamics of physiological and biochemical response to toxic effects, as well as to specify the concepts of homeostasis, stress, and mechanisms of adaptation to the effects of foreign chemical compounds. The result of this integration can be a complete mapping of the “toxicity pathways” and creating a “toxome”—a holistic understanding of the mechanisms of action of substances and the relationship between the dose, time, and consequences of exposure [212, 244]. These two scientific directions are almost ready to converge due to development of common algorithmic and computational models and tools. Interestingly, the computational collaboration between different institutions is now considered as one of the most difficult problems, because existing approaches cannot manage with the growing volume of heterogeneous data and provide an adequate computational environment for consistent scientific analysis [245].

The endothelial interface in the tissues and the ROS interface in the cells represent the two principal boundaries, cellular and molecular, respectively, that integrate the interior and exterior of the living body. It is now evident that ROS in general and H_2_O_2_ in particular serve as the trigger system of ECs and other cells, providing Ca^2+^-dependent regulation of metabolism with a row of other auxiliary molecules and channels. Not breaking the signaling and metabolic pathways, ROS implement the “orthogonal regulation” of metabolism through modulation of kinetic characteristics of biochemical reactions [239]. Redox modulation is specifically localized and depends on the size and redox potential of ROS or other oxidants (electrophile); its concentration, and availability of the targets, consisted mainly of amino acids [246]. It is important to note here that the so-called antioxidants are perhaps the most ambiguous and speculative group of dietary supplements. Vitamins C and E are used by many people, who believe that high dosages of antioxidants improve health and resistance to disease. These assumptions are, however, generally not supported by scientific data. Antioxidant supplementation has been demonstrated to exert adverse effects, especially on adaptation to intense training [247, 248]. These antioxidants can actually reduce the level of oxidative stress molecules, though this “improvement” often has no correlation with adaptation to strenuous exercise, and many antioxidant-based clinical trials and therapeutic interventions have been largely disappointing in their therapeutic benefit [249, 250]. At the same time, some natural metabolites (pyruvate, ethylpyruvate, fructose-1,6-bisphosphate, etc.) or nutraceuticals taken exogenously as food additives or separately can effectively neutralize oxidative stress, improve health, and even enhance adaptation to physical stress [250, 251]. An important prerequisite of the new concept in redox biology/medicine is the use of compounds that has been termed “pathologically activated therapeutics,” that is, relatively small molecules that are chemically converted to their active form by the very oxidative stress which they are intended to combat [252]. Sulfides, disulfides, and other organosulfur compounds (OSC) are widely present in our bodies and the natural environment. They can interact with or neutralize ROS and determine the thiol/disulfide redox states in signaling and sensing, thus maintaining our bodies healthy or contributing to development of diseases [253]. On the other hand, some proteins are not as readily formed when the redox state of the cell is insufficiently oxidative, and the famous James D. Watson even suggested the type 2 diabetes to be a redox disease, which could be treated by moderate physical exercise [254]. Exercise training can optimize the redox environment by enhancing the capacity of cells to neutralize ROS [255]. Disulfide bridges that are formed during the posttranslational modifications of proteins are highly important in keeping their tertiary structure and maintaining their functional activity. Disulfide bond formation is possible due to oxidative potential existing in specific cellular compartments, and it was wittily noted by Bardwell that “the long-running race to find the source of oxidizing potential for disulfide bond formation is over, and the winner is one of the first contestants to enter: oxygen” [256]. Nevertheless, oxygen is not always available in plenty amounts, for example, in ischemic states. In such cases, some artificial or natural electrophiles (electron acceptors) could be helpful, and this property has been exploited for elaborating a new type of therapeutics with the help of OSC, polyphenols, and other nutraceuticals [250, 253]. Nutraceuticals could serve not only as electrophiles, but also as a kind of “pro-electrophilic drugs” that become electrophilic in response to oxidation with subsequent activation of the Keap1/Nrf2/ARE transcription pathway and synthesis of endogenous antioxidant “phase 2” enzymes [252]. Many nutraceuticals have multiple molecular and functional targets in humans and animals, and at least two consequences follow from this fact: (1) experimental animal and *in vitro* studies can hardly be overestimated; (2) studies of nutraceuticals should be based on the multitarget drug approach, according to which focus should be placed on selecting molecular targets, judging their relevance, and estimating safety concerns [257]. For example, many natural products are agonists of PPAR*γ* and/or PGC-1*α*, and the bioactivity pattern of the active compounds and plant extracts warrants future research regarding their therapeutic potential and the possibility to modulate PPAR*γ*/PGC-1*α* activation by dietary interventions or food supplements [258]. Some researchers consider OSC as ideal nutraceutical agents as they can serve not only as direct antioxidants trapping electrons, but also have nonantioxidant effects such as antiplatelet, fibrinolytic, anti-inflammatory, immunomodulatory, antiageing actions, and so forth [259].

Presently, diet quality indices that embrace overall diet quality in contrast to single nutrients draw increased attention. This is especially relevant to the state of endothelium and vascular diseases. There is a strong association between food patterns (FPs) and endothelial biomarkers. Healthy FPs (higher intakes of fruits and vegetables) have a beneficial effect on endothelial functions, as estimated by the levels of biomarkers such as CRP, sICAM-1, sVCAM-1, and E-selectin molecules. Westernised FPs (abundant in meat, sweets, fried foods, and refined grains) correlated with biomarkers of inflammation and atherogenesis [260]. Apart from food and in contrast to the abovementioned “antioxidants,” moderate aerobic exercises positively affect both redox state biomarkers and cognitive abilities in older adults [261]. To consider another complicated problem, peripheral neuropathy is among the prevailing neurologic conditions encountered by physicians and toxicologists [192, 262]. Again, there are three distinct challenges for physicians in caring for patients with peripheral neuropathy: (1) how to efficiently and effectively screen a patient for peripheral neuropathy, (2) how to clinically stratify patients, and (3) how to treat the symptoms [262]. Limitations in study designs together with the large variability in surrogate markers of vascular and other tissue damage have reduced the strength of the evidence base [263]. Microvascular lesion in diabetic peripheral arterial disease still cannot be resolved by current surgical and interventional techniques, though ECs have the therapeutic potential to cure microvascular lesion through rather exquisite and elaborate transcatheter arterial infusion of autologous CD133(+) cells [264]. One of the obstacles affecting this process is that stem cells retreived from eldery patients tend to be presenescent or already in senescence. Therefore, *in vitro* manipulations can cause the aged stem cells to undergo massive *in vivo* apoptosis after transplantation [265]. At the same time, endothelial progenitors can be mobilized by noninvasive “technique”—yes, with nutraceuticals: Stem-Kine complex augmented hemopoietic and endothelial progenitors (CD133+ and KDR-1/CD34+) just the next day after beginning of administration [266]. Moreover, moderate exercises actually improve vascular function in patients with type 2 diabetes [267], thus giving us confidence in elaborating more natural approaches to solve the problems of vascular health and disease.

Now, we have to finalize and feel obliged to offer some advice, so we will try. In order to prevent or even cure a disease, we need to repair blood vessels in advance or in parallel. For this, we need first to acquire adequate information through adequate diagnostics, which would comprise monitoring the state of endothelium with specific markers/biomarkers. Inter alia, this would help to completely remove the damaging factors. Then, we need to apply natural means (nutraceuticals and physical activity), which would not help to stop flows completely, but to tune their kinetics and harmonize the metabolic and signaling pathways due to soft power, influencing the two principal interfaces: endothelial cells throughout the body and ROS signaling in these and many other cells throughout the body. These advises are not only for “external consumption,” they are the essense of our own perspectives in science.

When something is rotten in a state, one can differently sense it being outside or inside of the state. Supplying adequate information, sometimes simply through mass media, can greatly help people to rationally exploit the natural, material, and financial resources of the state, according to its place on Earth, history, and culture. It is cheap and cheerful. Application of nutraceuticals and physical activities for curing the rotten state of endothelium would greatly help all the tissues and organs, according to their place in the body, ontogenetic features, and functional traits. This is cheap and cheerful, too.

## Figures and Tables

**Table 1:** Endothelial cell markers (modified from https://www.rndsystems.com/research-area/endothelial-cell-markers).

Endothelial cell marker	Function
CD13/APN	CD13/aminopeptidase N is a transmembrane peptidase that is induced in the vasculature of solid tumors and is a potent angiogenic regulator. CD13 functions as a novel modulator of signal transduction and cell motility via its influence on specific plasma membrane organization, thus regulating angiogenesis [25].

CD29/integrin *β*1	Integrin *β*1 participates in endothelial sprouting, though negatively regulates proliferation of ECs. In maturing vessels, integrin *β*1 is needed for proper localization of VE-cadherin and cell-cell junction integrity; it is required for the formation of nonleaky blood vessels [26]. Integrin *β*1 is necessary for the interactions between cardiomyocytes and endothelial cells, thus regulating cardiac myocyte reorganization [27].

CD31/PECAM-1	CD31, known as PECAM-1 (platelet/endothelial cell adhesion molecule 1), is a heavily glycosylated transmembrane homophilic adhesion protein that is highly expressed on endothelial cells and is required for migration of leukocytes, playing a key role in removing aged neutrophils from the body. The extracellular domain of CD31 is released during endothelial cell apoptosis. This fragment circulates in the serum of patients suffering from myocardial infarction, acute ischemic stroke, and multiple sclerosis [28].

CD34	CD34 is a transmembrane phosphoglycoprotein, first identified on hematopoietic stem and progenitor cells. Cells expressing CD34 are found in the umbilical cord and bone marrow as endothelial progenitor cells, a subset of mesenchymal stem cells, hematopoietic cells, and ECs of blood vessels and pleural lymphatic vessels. The presence of CD34 on nonhematopoietic cells in various tissues has been linked to progenitor and adult stem cell phenotypes [29].

CD36/SR-B3	CD36 is known as scavenger receptor class B member 3 (SR-B3), thrombospondin receptor, collagen receptor, platelet membrane glycoprotein IV (GPIV), GPIIIb, and fatty acid translocase (FAT). Upon ligand binding, CD36 transduces signals that mediate a wide range of proinflammatory cellular responses. For example, amyloid-*β*_1–40_ (A*β*) activates the innate immunity receptor CD36 leading to production of superoxide anions via NADPH oxidase [30].

CD39/ENTPD1	CD39 (ENTPD1) is an ectonucleotidase most abundantly expressed on the surface of ECs and to a lesser degree on the surface of platelets and leukocytes. CD39 catalizes extracellular dephosphorylation of ATP to ADP and AMP. CD39 molecules are liberated from the coronary vascular endothelium by ischemia-reperfusion, and levels of circulating ectonucleotidase may reflect the severity of ischemic vascular injury [31]. Attenuation or lack of CD39 activity is associated with vascular dysfunction and remodeling in pulmonary arterial hypertension [32], decreased hepatic regeneration, and failure of vascular reconstitution [33].

CD44	CD44 expression in endothelial colony-forming cells (ECFCs) regulates neurovascular trophic effect [34]. ECFCs are adult endothelial progenitor cells (EPCs), which can differentiate and participate in regeneration of ECs. They reside in vasculature and can migrate to places of injury as a form of circulating ECs [35]. ECFCs play an important role in vascular healing after injury and developmental angiogenesis. Interestingly, overexpression of CD44v (8–11) stabilizes the cystine/glutamate antiporter to increase cysteine and GSH levels in cancer stem cells [36].

CD47	Endothelial CD47 molecules participate in intracellular calcium mobilization, increased permeability, and activation of Src and AKT1/PI3K in brain microvascular ECs [37]. Activation of these signaling pathways results in cytoskeleton reorganization and cadherin phosphorylation, which are necessary steps for T-cell transendothelial migration. The overlapping effect of CD54 (ICAM-1) and CD47 suggests their involvement in different steps of the diapedesis process.

CD54/ICAM-1	ICAM-1 (intercellular adhesion molecule-1) is a transmembrane protein that is upregulated on endothelial and epithelial cells at sites of inflammation. It mediates the vascular adhesion and paracellular migration of leukocytes with activated LFA-1 (CD11a/CD18) and Mac-1 (CD11b/CD18). Soluble ICAM-1 participates in angiogenesis being an indicator of EC activation or damage. Elevated levels of soluble ICAM-1 are linked to oxidative stress, hypertension, cardiovascular disease, type 2 diabetes, organ transplant dysfunction, increased abdominal fat mass, liver disease, certain malignancies, and sepsis [38].

CD61/integrin *β*3	CD61 (integrin *β*3) is a protein taking part in platelet aggregation, being considered for a long time as a marker only for this kind of cells, and then found on endothelial cell surface bound to some proteins, in particular disulfide isomerase [39].

CD62E/E-selectin	E-selectin (endothelial leukocyte adhesion molecule-1 (ELAM-1), CD62E) is one of the three members of the selectin family (E-selectin, L-selectin, and P-selectin), which is transiently expressed on ECs in response to IL-1*β* and TNF-*α*.

CD62P/P-selectin	Also known as GMP-140, PADGEM, and LECAM-3; a transmembrane adhesion protein that is upregulated on activated platelets and vascular endothelial cells. P-selectin mediates the initial interaction of circulating leukocytes with activated endothelial cells that produces a characteristic rolling of the leukocytes on the endothelium. It is therefore critical for leukocyte extravasation at sites of inflammation and leukocyte involvement in thrombus formation at sites of vascular injury.

CD80/B7-1	Under certain conditions, ECs can serve as antigen presenting cells by expressing both MHC class I and class II molecules [17]. For example, hepatic sinusoidal ECs have the ability to express the costimulatory adhesion molecules CD80 (B7-1) and CD86 (B7-2), expression of which can be increased by ischemia/reperfusion of the rat liver. Also, CD80, CD86, and ICAM-1 molecules were upregulated in the glomerular and peritubular ECs after ischemia/reperfusion [40].
CD86/B7-2	

CD93/C1qR1	C1qR1 is also known as C1qRp, collectin receptor, and AA4 antigen. It is a type I transmembrane glycoprotein found not only on ECs, but also on hematopoietic progenitor cells, platelets, and cells of myeloid origin. C1qR1 molecules mediate phagocytosis by monocytes and macrophages upon interaction with soluble defense collagens, such as C1q, MBL, and SP-A.

CD102/ICAM-2	ICAM-2 molecules are abundantly expressed on vascular ECs and lymphohematopoietic cells. They participate in T cell aggregation, NK cytotoxicity, and NK cell migration.

CD105/endoglin	Endoglin is a transmembrane type III receptor for TGF-*β* superfamily ligands and plays an important role in smooth muscle differentiation, angiogenesis, and neovascularization. It is highly expressed on proliferating vascular ECs, chondrocytes, and syncytiotrophoblasts of term placenta. Human endoglin haploinsufficiency can cause the vascular disorder, hereditary hemorrhagic telangiectasia type I. Elevated levels of antiangiogenic soluble endoglin contribute to pathogenicity in preeclampsia.

CD106/VCAM-1	Vascular cell adhesion molecule-1 is a transmembrane molecule that mediates the adhesion of immune cells to the vascular endothelium during inflammation. It binds to several integrins including *α*4/*β*1 (VLA-4), α4/β7, α9/β1, and αD/β2. It is expressed constitutively in the bone marrow where it regulates T cell, B cell, and hematopoietic progenitor cell migration. A soluble form VCAM-1 can promote monocyte chemotaxis.

CD112/nectin-2	Nectins are type I transmembrane glycoproteins that are calcium-independent Ig-like CAMs. Due to the sequence homology, they were designated as poliovirus receptor-related (PRR) proteins, though do not bind poliovirus. Nectin-1 (CD111, PRR1, herpes virus entry mediator C, or HVEC), nectin-2 (CD112, PRR2, and HVEB), and nectin-3 (PRR3) are found in adherens junctions on neurons, ECs, epithelial cells, and fibroblasts.

CD117/c-Kit	CD117/c-Kit is a receptor with tyrosine kinase activity that binds stem cell factor; it is a marker of certain types of hematopoietic progenitors in the bone marrow (hematopoietic stem cells, multipotent progenitors, and common myeloid progenitors). Also, its expression is a distinguishing feature of hemogenic ECs, relative to nonblood-forming ECs [41].

CD121a/IL-1 RI	IL-1 RI, also known as the type 1 IL-1 receptor and CD121a, is a transmembrane protein in the Toll/IL-1 R (TIR) superfamily. IL-1 RI binds the pleiotropic cytokines IL-1*α* and IL-1*β*, plus the IL-1 receptor antagonist (IL-1 Ra). Signal transduction requires complex formation with IL-1 R AcP/IL-1 R3. This complex recruits the adaptor protein MyD88, to initiate signaling in the NF-κB pathway. IL-1 RI is expressed predominantly by T cells, fibroblasts, and ECs and mediates acute phase inflammatory responses including fever.

CD141/thrombomodulin/BDCA-3	Thrombomodulin (TM), also known as CD141 and BDCA3, is a transmembrane expressed on vascular ECs, arterial smooth muscle cells, monocytes, and macrophages. It binds thrombin and enhances the thrombin-mediated activation of the anticoagulant protein C and the antifibrinolytic TAFI/carboxypeptidase B2. Thrombomodulin also inhibits the ability of thrombin to activate several procoagulant proteins (e.g., fibrinogen, factor V, factor XIII, and PAR-1). Soluble fragments of thrombomodulin are elevated in the serum, urine, and synovial fluid during coagulation disorders, inflammation, and organ failure.

CD142/coagulation factor III/tissue factor/thromboplastin	Tissue factor is an integral membrane protein, which has been shown to be produced by several cell types, including ECs. It serves as a cofactor of coagulation factor VII.

CD143/ACE	ACE and ACE2 are cell surface proteases, which maintain blood pressure homeostasis and fluid salt balance, mainly due to generation of angiotensin II and inactivation of bradykinin. Also, ACE activities play roles in immunity, reproduction, and neuropeptide regulation.

CD144/VE-cadherin	Vascular endothelial (VE)-cadherin is a specific endothelial adhesion molecule located at junctions between ECs. VE-cadherin-mediated adhesion is important for the control of vascular permeability and leukocyte extravasation. Also, VE-cadherin participates in cell proliferation, apoptosis, and modulates vascular endothelial growth factor receptor functions [42].

CD146/MCAM	CD146 is also known as the melanoma cell adhesion molecule (MCAM) or cell surface glycoprotein MUC18. CD146 is currently used as a marker for ECs. MCAM functions as a receptor for laminin alpha 4, a matrix molecule expressed within the vascular wall by ECs, SMCs, and pericytes. Downregulation of MCAM accelerates cellular senescence in human umbilical cord blood-derived mesenchymal stem cells [43]. On the other hand, MCAM is an adverse prognostic factor in uterine sarcoma [44].

CD147/TRA-1-85	The TRA-1-85 antigen, or Oka blood group antigen, is a specific epitope of the protein basigin, also known as EMMPRIN and CD147.

CD151	CD151 is a tetraspanin superfamily glycoprotein expressed by ECs, epithelial cells, megakaryocytes, and platelets. It interacts with other tetraspanins and integrins, such as α3/β1, α6/β1, α6/β4, and α7/β1. CD151 plays a role in cellular adhesion, migration, and platelet activation.

CD160	CD160 is a GPI-anchored glycoprotein with one Ig-like V-type domain, found mainly on a subpopulation of cytolytic T cells and NK cells. CD160 serves as a receptor for MHC class I and related molecules. When expressed on ECs, CD160 plays a role in antiangiogenic signaling and apoptotic cell death.

CD201/EPCR	EPCR (the endothelial protein C receptor) is a transmembrane glycoprotein expressed on ECs. EPCR inhibits thrombosis through its interactions with protein C, activated protein C (aPC), and coagulation factors VII and VIIa. It enhances the activation of protein C in response to complexes of thrombin-thrombomodulin. A soluble form of EPCR inhibits the anticoagulant activity of APC. EPCR binds to CD11b/CD18 (Mac-1) on monocytes and mediates monocyte adhesion to the vascular endothelium. In addition, it binds to the antigen receptor on gamma/delta T cells, promotes hematopoietic stem cell retention in the bone marrow, and contributes to malaria pathogenicity through binding surface proteins on plasmodium.

CD213a/IL-13R alpha 1	Two members of the type 5 subfamily of type I cytokine receptors also serve as receptors for IL-13, which bind to IL-13 R α1 (CD213a1, also known as NR4) with low affinity, then forming a high affinity receptor together with the IL-4 R alpha chain and causing downstream STAT6 activation. On the other hand, IL-13 can bind IL-13 R alpha 2 (CD213a2) with high affinity and subsequent TGF-β production, though without activation of STAT6.

CD248/endosialin	Endosialin, also known as tumor endothelial marker 1 (Tem1), is a 165 kDa transmembrane O-glycosylated protein that contains one C-type lectin, one sushi, one EGF-like domain, and a mucin-like stalk in its extracellular domain (ECD). It is expressed on activated perivascular and stromal cells in embyronic and tumor neovasculature but is downregulated in quiescent vasculature. Endosialin regulates pericyte proliferation, migration, and adhesion to matrix fibronectin and collagens I and IV.

CD309/VEGFR2/KDR/Flk-1	VEGFR2 (vascular endothelial growth factor receptor 2) is a transmembrane receptor tyrosine kinase that mediates the angiogenic effects of VEGF-A and VEGF-C. It is expressed primarily on vascular ECs and endothelial cell progenitors. It is also expressed on endometrial epithelium, hematopoietic stem cells, liver sinusoidal ECs, Sertoli cells and Leydig cells, platelets and megakaryocytes, sensory and autonomic neurons, Schwann cells, Muller glial cells, retinal progenitors, and osteoblasts. An increase in CD309 expression leads to increase of endothelial permeability in microvascular bed [45].

ADAMs 8, 9, 10, 12, 15, 17, and 33	The disintegrin and metalloproteinases ADAM10 and ADAM17 serve as principal regulators of cytokines, growth factors, and adhesion molecules, through proteolytic shedding on the cell surface [46]. ADAMs 12 and 17 expressed in ECs are responsible for the impairment of vascular barrier by hypoxia, possibly through proteolysis of claudin-5, a tight junction molecule, on EC membranes [47].

ADAMTS-13	ADAMTS-13 (A disintegrin-like and metalloprotease with thrombospondin type 1 repeats-13) is a zinc-containing metalloprotease enzyme that cleaves von Willebrand factor. It is produced in liver stellate cells and ECs and is present in platelets. It is also produced in kidney podocytes with subsequent deposition in the glomerular basement membrane, thus preventing clot formation. ECs have a major contribution to the bulk production of ADAMTS-13 in the body [1].

ADAMTS-18	ADAMTS-18 is a member of a disintegrin and metalloproteinase with thrombospondin motifs (ADAMTS) family of proteases, participating in angiogenesis and coagulation; dysregulation of these enzymes leads to inflammation, cancer, arthritis, atherosclerosis, and other diseases. Endothelial cell ADAMTS-18 secretion is enhanced by thrombin. Platelet aggregates can be destroyed by the C-terminal ADAMTS-18 fragment [48].

CXCL16	Transmembrane CXC chemokine ligand 16. Eryptotic erythrocytes adhere to ECs of the vascular wall in part by interaction of phosphatidylserine exposed at the erythrocyte surface with endothelial CXCL16 [49].

DCBLD2/ESDN	DCBLD2 (discoidin, CUB, and LCCL domain containing 2), also known as ESDN (endothelial and smooth muscle cell-derived neuropilin-like) and CLCP1, has structural similarities to neuropilins, VEGF receptors, and semaphorins and participates in cell motility and metastasis.

Endomucin	Endomucin (endothelial sialomucin; also endomucin-1/2 and mucin-14) is an 80–120 kDa glycoprotein member of the endomucin family of proteins. It is expressed on ECs and function as either a pro- or antiadhesive molecule, depending on its glycosylation pattern.

ESAM	ESAM (endothelial cell-selective adhesion molecule) is an EC-associated member of the CTX subgroup of the Ig superfamily. It is associated with tight and adherens junctions and also modulates transendothelial cell migration along with FGF-2.

FABP	Fatty acid-binding proteins (FABPs) are small cytoplasmic lipid-binding proteins, which can bind free fatty acids, cholesterol, and retinoids, and are involved in intracellular transport of lipids. Along with other biomarkers, circulating FABPs are used as indicators of tissue damage. Hypoxia affects expression of FABP in ECs [50].

IgG (immunoglobulin G)	IgGs on HUVECs behave as Ig-producing immune cells. Under the stimulus of external IgGs, some secretory pathways are also activated in HUVECs together with the expression of FcRn, which is associated with newly synthesized IgGs, thus forming complexes to be secreted [51].

Integrin *α*4*β*1/VLA-4	Integrin *α*4*β*1 (VLA-4) and VCAM-1 ensure close intercellular adhesion between ECs and pericytes, which is required for blood vessel formation. Integrin *α*4*β*1 is expressed by proliferating ECs, and VCAM-1 is expressed by proliferating pericytes. Antagonists of this interaction block the adhesion of perivascular cells to proliferating ECs, thereby inducing apoptosis of ECs and pericytes and inhibiting neovascularization [52].

KLF4	Krüppel-like factor 4 (KLF4) is a transcription factor, which is a central regulator of sprouting angiogenesis via regulating Notch signaling pathway [53]. Endothelial KLF4 is renoprotective and mediates statin-induced protection against ischemic AKI by regulating the expression of cell adhesion molecules and concomitant recruitment of inflammatory cells [54].

LYVE-1	Lymphatic vessel endothelial hyaluronan (HA) receptor-1 (LYVE-1) is a 60 kDa type I transmembrane glycoprotein that is a member of the link protein superfamily. It modulates cell behavior and exerts activity during development, tissue remodeling, and disease. It is a marker of lymphatic ECs and is also expressed on hepatic sinusoidal ECs. To a lesser degree, LYVE-1 is found on Kupffer cells, the islets of Langerhans, cortical neurons, and renal epithelium.

Notch	Notch signaling is an evolutionary conserved pathway critical for cardiovascular development. The contribution of Notch signaling to the homeostasis of the adult vasculature has emerged as a novel paradigm, which suggests that this pathway is sensitive to environmental factors, including inflammatory mediators and diet-derived by-products, including glucose and lipid metabolites [55].

Podocalyxin	Podocalyxin (also known as podocalyxin-like protein 1/PODXL or PCLP1) is a sialoglycoprotein that is structurally related to CD34. Podocalyxin is abundantly expressed in embryonic stem cells and also is a marker of hemangioblasts, the common precursors of hematopoietic and ECs.

Podoplanin	Podoplanin, also known as T1 alpha and Aggrus, is a mucin-type transmembrane glycoprotein with extensive *O*-glycosylation. It is expressed by lymphatic ECs, as well as by nonendothelial cells in some tissues. Podoplanin participates in regulation of lymphatic vascular formation and platelet aggregation.

RLIP76/RALBP1	RLIP76 (Ral-interacting protein of 76 kDa), also known as RalBP1 (Ral-binding protein 1), is an ATP-dependent transporter of electrophile-glutathione conjugates [56].

Stabilin-1 and stabilin-2	Stabilin-1 and stabilin-2 are type I transmembrane members of a family of fasciclin-like hyaluronan (HA) receptor homologs, expressed on sinusoidal ECs and macrophages.

TEM8/ANTXR1	Tumor endothelial marker 8 (TEM8) is one of eight TEM gene products that are associated with tumor angiogenesis. TEM8 and CMG2 (capillary morphogenesis gene 2) are type I transmembrane proteins with an extracellular von Willebrand factor type A domain. They are members of an anthrax toxin receptor family. TEM8 is highly expressed in tumor vasculature and may function as an adhesion molecule during capillary tubulogenesis.

THSD1	THSD1 (thrombospondin type-1 domain-containing protein 1), also known as transmembrane molecule with thrombospondin module (Tmtsp), is a type I transmembrane protein of 95 kDa. It is highly expressed in hematopoietic stem cells and progenitor cells. Also, THSD1 is abundantly expressed on ECs, with the highest expression in the lung. THSD1 is involved in the regulation of vasculogenesis and/or angiogenesis.

Tie-1 and Tie-2	Tie-1/Tie and Tie-2/Tek are receptor tyrosine kinases which have two immunoglobulin-like domains flanking three EGF-like domains, three fibronectin type III-like repeats in the extracellular region, and a split tyrosine kinase domain in the cytoplasmic region. The Tie-2 receptor is an essential regulator of vascular barrier function. Angiopoietins 1 and 2 are the principal ligands of Tie-2, which can exert opposite effects on this receptor [57].

TNAP	Tissue nonspecific alkaline phosphatase (TNAP) is localized at ECs of brain blood vessels and neuronal membranes, inducing neuronal toxicity via tau dephosphorylation. This function can play a role in the neuronal loss observed in Alzheimer's disease. TNAP level is increased in blood plasma during cerebrovascular diseases and after brain injury [7, 58].

TNF RII/TNFRSF1B	TNF RII (tumor necrosis factor receptor II), also known as TNFRSF1B, p75/p80, and CD120b, is a widely expressed receptor for membrane-associated TNF-α and lymphotoxin-α. Its activation initiates proinflammatory and prosurvival responses via NF-*κ*B-dependent signaling pathways, although it may also induce apoptosis.

VE-cadherin	The cadherin superfamily consists of a large number of cell surface glycoproteins with cadherin repeats, upon which a Ca^2+^-dependent cell-cell adhesion depends. VE-cadherin is a major endothelial adhesion molecule controlling cellular junctions and blood vessel formation [42].

VE-statin	VE-statin is a 41 kDa secreted glycoprotein, which is a member of a rather large family of EGF-like domain-containing proteins. VE-statin is a marker of embryonic ECs and is also expressed in ECs of adults.

VG5Q	VG5Q, also known as AGGF1, is associated with Klippel-Trenaunay syndrome (KTS), a congenital vascular morphogenesis pathology. VG5Q is expressed by vascular ECs in many tissues. It can be secreted, thus promoting proliferation of the neighboring ECs.

VWF	Von Willebrand factor (VWF) is a glycoprotein participating in blood coagulation. It is released from Weibel–Palade bodies (WPB) of ECs and exhibits a binding site for factor VIII (FVIII) and also for heparin. VWF size and function are regulated by protease ADAMTS-13, and disturbance of this function can lead to thrombotic thrombocytopenic purpura. Also, endothelial VWF regulates angiogenesis [59].

## Abbreviations

*α*-SMA: Alpha-smooth muscle actin
A*β:*: Amyloid-*β*_1–_40
ACE: Angiotensin-converting enzyme
AChE: Acetylcholinesterase
AD: Alzheimer's disease
ALT: Alanine transaminase
Angpt: Angiopoietin
Ang: Angiotensin
AST: Aspartate transaminase
BBB: Blood-brain barrier
BMP-7: Bone morphogenetic protein 7
CAM: Cell adhesion molecules
CECs: Circulating endothelial cells
CNS: Central nervous system
CRP: C-reactive protein
CVD: Cardiovascular disease
CWDF: Chemical weapon destruction facility
DAG: Diacylglycerol
DFP: Diisopropyl fluorophosphate
DGK*α:*: Diacylglycerol kinase alpha
ECs: Endothelial cells
EDRF: Endothelium-dependent relaxing factor
EGF: Epidermal growth factor
EMPs: Endothelial microparticles
EndoMT: Endothelial-to-mesenchymal transition
EPC: Endothelial progenitor cells
ER: Endoplasmic reticulum
ET-1: Endothelin-1
FAT: Fatty acid translocase
FGF: Fibroblast growth factor
FPs: Food patterns
GGT: *γ*-glutamyl transferase
GSK: Glycogen synthase kinase
HIF-1*α:*: Hypoxia-inducible factor-1*α*
HUVECs: Human umbilical vein endothelial cells
ICAM: Intercellular adhesion molecule
Ig: Immunoglobulin
LDH: Lactate dehydrogenase
LDL: Low-density lipoprotein
LFA-1: Lymphocyte function-associated antigen 1
MAPK: Mitogen-activated protein kinase
mTOR: Mechanistic target of rapamycin
NOS: NO synthase
NOX: NADPH oxidase
OP-1: Osteogenic protein-1
OPs: Organophosphates
OSC: Organosulfur compounds
ox-LDL: Oxidized low-density lipoprotein
PA: Phosphatidic acid
PECAM-1: Platelet EC adhesion molecule-1
PGC-1*α:*: Peroxisome proliferator-activated receptor *γ* coactivator 1*α*
PI3K: Phosphoinositide 3-kinase
PKC: Protein kinase C
PMA: Phorbol 12-myristate 13-acetate
PPAR*γ:*: Peroxisome proliferator-activated receptor *γ*
Prxs: Peroxiredoxins
PTK: Protein tyrosine kinase
RAS: Renin-angiotensin system
RFK: Riboflavin kinase
ROS: Reactive oxygen species
SIRTs: Sirtuins
SOD: Superoxide dismutases
TGF-*β:*: Transforming growth factor-*β*
TNAP: Tissue-nonspecific alkaline phosphatase
TNF-*α:*: Tumor necrosis factor alpha
t-PA: Tissue plasminogen activator
TRP: Transient receptor potential
VE-cadherin: Vascular endothelial cadherin
VEGF: Vascular endothelial growth factor
VEGFR: VEGF receptor
VE-PTP: Vascular endothelial protein tyrosine phosphatase
VVOs: Vesiculo-vacuolar organelles
VWD: VWF disease
VWF: Von Willibrand factor.
